# Microglia rely on SYK signalling to mount neuroprotective responses in models of Alzheimer's disease and multiple sclerosis

**DOI:** 10.1002/ctm2.1178

**Published:** 2023-01-11

**Authors:** Hannah Ennerfelt, John R. Lukens

**Affiliations:** ^1^ Department of Neurology Stanford University Palo Alto CA USA; ^2^ Center for Brain Immunology and Glia (BIG) Department of Neuroscience University of Virginia Charlottesville VA USA

**Keywords:** Alzheimer's disease, amyloid beta, microglia, multiple sclerosis, myelin debris, neurodegenerative disease, neuroimmunology

1

After a generation of failed therapies, microglia have captivated scientists searching for unexplored avenues to treat neurodegenerative diseases. The last decade of research has unveiled a remarkable capacity for microglia to alter the trajectory of neurodegeneration through the interaction of these brain‐resident immune cells with disease pathology and other disease‐associated cell types in the brain. Emerging from this collective work is the idea that microglia can play both neuroprotective and deleterious roles in neurological disease pathogenesis. However, what accounts for the acquisition of damaging versus beneficial functions by microglia is still a matter of great debate in the field. Despite this, recent advances have begun to uncover some of the major transcriptional networks and effector mechanisms that underpin the ability of microglia to impact neurodegenerative disease susceptibility and progression. Unearthing the processes by which microglia can modify pathology in neurodegenerative diseases such as Alzheimer's disease (AD) and multiple sclerosis (MS) will likely hold key therapeutic insights for successful interventions by clinicians.

Although numerous mechanisms have been proposed to date, it has become increasingly clear that the ability of microglia to corral and phagocytose neurotoxic material, including amyloid beta (Aβ) in AD and damaged myelin in MS, is essential for them to exert their neuroprotective effects in degenerative disease.^1^ Consistent with this idea, recent studies demonstrate that the TREM2 receptor is deployed by microglia to coordinate both the containment and phagocytosis of Aβ and dead cells in an effort to limit the toxicity of these pathologies to surrounding neurons.^2^, ^3^ The CD33 receptor, on the other hand, has been shown to inhibit the ability of microglia to phagocytose Aβ.^4^ In‐line with this, gain‐of‐function mutations in CD33 have been strongly linked with AD risk in humans.^5^

Age is the greatest risk factor for AD, and it has also been proposed that aging‐related defects in phagocytosis contribute to the increased risk of developing neurodegenerative disease. This has motivated many groups to perform screens in the search of the molecular players that underlie age‐associated decline in microglial phagocytosis. In one such study, it was shown that the receptor CD22 hinders microglial phagocytic capacity during aging.^6^ Furthermore, relevant to both AD and MS, this study reported that antibody‐based blockade of CD22 provides an effective strategy to boost the phagocytosis of both Aβ and myelin debris. Although great strides have recently been made in defining some of the surface receptors that modulate microglial biology in neurodegenerative disease, the identity of the key intracellular signalling molecules exploited by microglia to regulate their neuroprotective functions is currently less well understood.

Tremendous efforts in recent years have also been paid to defining how microglial responses evolve during the course of neurodegenerative disease in hopes of unearthing key transcriptional signatures and molecular players that influence disease progression. Emerging from these collective studies is the notion that microglia take on a neuroprotective disease‐associated microglia (DAM) phenotype during neurodegenerative disease progression, and that this transformation is meant to equip them with the machinery needed to properly contain and dispose of neurotoxic material.^7^ This transition is characterized by the downregulation of homeostatic genes such as *Tmem119* and *P2ry12*, and the concomitant upregulation of DAM and neurodegenerative microglia (MGnD) factors that include *Lpl*, *Ccl6*, *Clec7a*, and *Cst7*.^7^, ^8^ Although this paradigm of microglial activation has been extensively studied and adopted in multiple models of neurological disease, we currently lack knowledge of the key signalling molecules that instruct this important transformation in microglia. However, in our recent work, we have identified spleen tyrosine kinase (SYK) as a novel intracellular coordinator of both phagocytosis and microglial transition from a resting state to a DAM/MGnD phenotype during neurodegeneration.

In our recently published study, we described neuroprotective microglial responses driven by the signalling molecule SYK in disease models of AD and MS.^9^ We demonstrated that SYK is critically involved in the acquisition of a DAM/MGnD phenotype in neurodegenerative disease and that disruption of this key signalling hub in microglia causes pronounced deficits in microglial activation, including defective Aβ phagocytosis and stunted proliferation in response to AD‐associated neuropathology. These defects in SYK‐coordinated microglial responses were further shown to cause exacerbated neuritic dystrophy, neuronal cell death, and cognitive impairment in the 5xFAD mouse model of AD. In‐line with these findings, an independent study published alongside our paper similarly showed that SYK signalling in microglia is required to support efficient containment and disposal of Aβ in the 5xFAD mouse model of AD.^10^ Both of our groups also revealed that boosting SYK activation can offer a robust strategy to enhance Aβ control in mouse models of AD.^9^, ^10^ More specifically, our collective studies demonstrated that activating the SYK‐associated receptor CLEC7A via injection with either anti‐CLEC7A antibodies or a natural, fungal‐derived CLEC7A ligand both offer effective interventions to limit amyloidosis in 5xFAD mice.

In addition, we found that the loss of SYK in microglia leads to more severe demyelinating neuroinflammatory disease in the experimental autoimmune encephalomyelitis model of MS. Using RNA‐sequencing, we revealed that SYK is centrally involved in microglial acquisition of a DAM/MGnD signature in response to both myelin debris and Aβ amyloidosis in models of MS and AD, respectively. Finally, by adopting cuprizone treatment as an alternative model of demyelinating disease, we observed that disruption of SYK signalling in microglia also leads to defects in microglial phagocytosis of damaged myelin and subsequent impairments in oligodendrocyte lineage cell repopulation upon disease resolution. The ability of microglial SYK to regulate other disease‐associated cell types relevant to regeneration, such as oligodendrocyte progenitor cells and oligodendrocytes, is an example of a potentially beneficial approach to target disease pathology and progression more comprehensively.

Taken together, our findings suggest that SYK is a key regulator of microglial DAM/MGnD transition and phagocytosis in response to both Aβ‐ and myelin‐driven neurodegenerative diseases (Figure 1). By continuing to expound on SYK signalling and its downstream regulators, more specific disease targets may be revealed, ultimately allowing for refined microglial interventions capable of curbing neurodegenerative disease progression.

**FIGURE 1:**
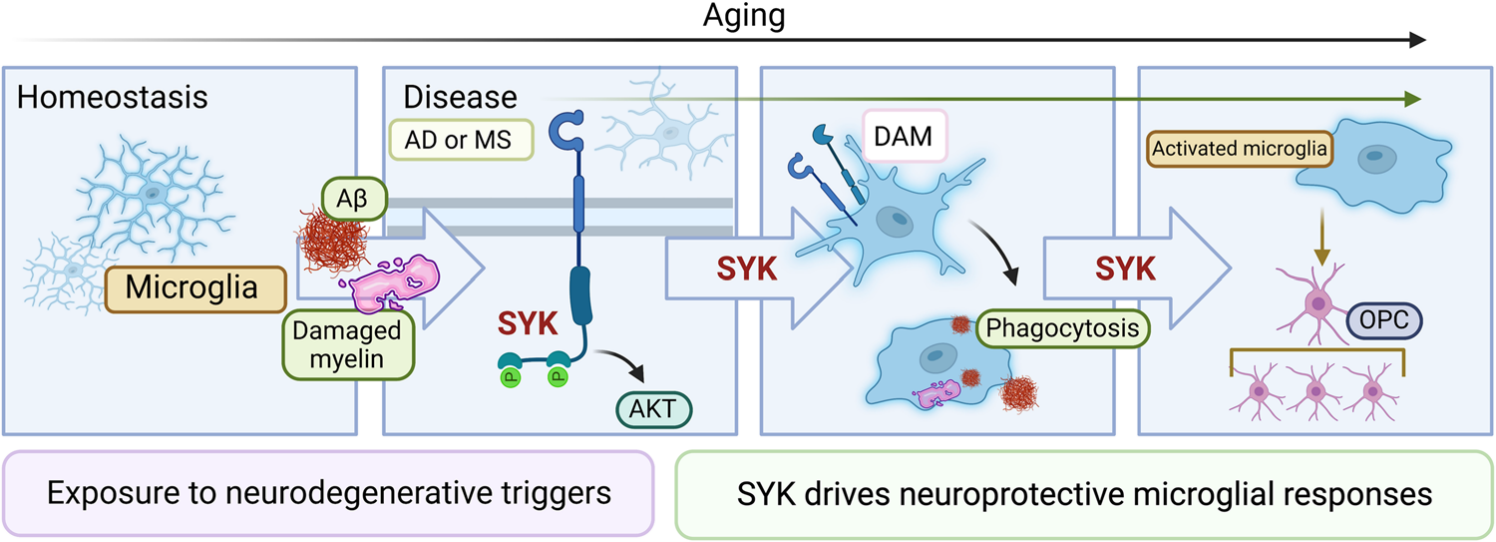
The microglial intracellular molecule known as spleen tyrosine kinase (SYK) is critically involved in driving neuroprotective microglial responses upon exposure to Alzheimer's disease (AD)‐ and multiple sclerosis (MS)‐associated neurodegenerative triggers. Amyloid beta (Aβ) and damaged myelin can prompt a transcriptional shift in microglia to become disease‐associated microglia (DAM) which can promote the upregulation of microglial phagocytic machinery to clear the pathology. Finally, SYK‐driven microglial activation regulates protective functions in other glial cells, such as oligodendrocyte progenitor cells (OPCs).

## CONFLICTS OF INTEREST

The authors declare no conflicts of interest
